# Book Reviews

**Published:** 1919-05

**Authors:** 


### BOOK REVIEW.

### Ultra Violet Rays in Dermatology.

Ultra Violet Rays in Dermatology. *Including the Evolution of Artificial Light Rays and Therapeutic Technique.* By Ralph Benstein, M. D., Philadelphia, Pa., Professor of Dermatology, Hahnemann Medical College, Philadelphia; Clinical Chief Skin Section, Hahemann Hospital Dispensary, Philadelphia, etc., etc., Author of "Elementary Dermatology," numerous brochures of skin diseases, etc. Illustrated. 162 pages. Lancaster, Pa.: Achey & Gorrecht, Publishers, 1918.

This little book is admirable for the simple and easy way in which what is ordinarily a difficult subject is handled. Its style is clear. Nothing is lost in authoritativeness by its compactness. It is a book that will surely interest those desiring to enter this fascinating field of therapeutics.

### Quarterly Medical Clinics.

Quarterly Medical Clinics. A Series of Consecutive Clinical Demonstrations and Lectures. By Frank Smithies, M. D., F. A. C. P., Associate Professor of Medicine, School of Medicine, University of Illinois; Gastro-Enterologist to Augustana Hospital; Medical Consultant to U. S. Marine Hospital; forml erly Gastro-Enterologist at Mayo Clinic, Fellow of The American Gastro-Enterological Association, etc., Augustana Hospital, Chicago. Published by Medicine and Surgery Publishing Company, Inc., Metropolitan Building, St. Louis. Single copies: $1.50, paper; $2.50 cloth. January, 1919.

As the initial number of Quarterly Medical Clinics, this offers a very attractive sample of what may be expected in future numbers. The book consists of a series of clinical lectures delivered to students of the School of Medicine, of the University of Illinois. These give a complete summary of the procedures of examination, diagnosis, and treatment indicated for each particular case presented. This method of study and presentation is imminently practical, making what is discussed of special value to the general practioner. Modern as well as the old tried methods are introduced. This number of Quarterly Medical Clinics discusses fifteen clinical cases.

